# Continuous PECS II block for postoperative analgesia in patients undergoing transapical transcatheter aortic valve implantation

**DOI:** 10.1186/s40981-017-0135-0

**Published:** 2017-12-12

**Authors:** Tomoharu Shakuo, Shinichi Kakumoto, Junya Kuribayashi, Katsunori Oe, Katsuhiro Seo

**Affiliations:** 1 Department of Anesthesiology, Showa University Northern Yokohama Hospital, 35-1 Chigasaki Chuou, Tsuzuki-ku, Yokohama-shi, Kanagawa 224-8503 Japan; 2 Department of Anesthesiology and Intensive Care Medicine, Kokura Memorial Hospital, 3-2-1 Asano, Kokurakita, Kitakyushu, 802-8555 Japan

## Abstract

It has been reported that PECS II block can alleviate postoperative pain following transapical transcatheter aortic valve implantation (TA-TAVI). However, the effectiveness of continuous PECS II block with catheterization has not yet been reported on the postoperative pain in patients undergoing TA-TAVI. We experienced two cases of TA-TAVI who received PECS II block with catheterization to manage postoperative pain. In the first case, a bolus injection for intraoperative pain and subsequent catheterization were performed before the implantation. However, the patient developed severe pain postoperatively in spite of the continuous block due to displacement of the catheter. In the second case, a bolus injection and the catheterization for the continuous block were performed before and after the implantation, respectively, which provided high-quality pain control. Continuous PECS II block may be useful to control perioperative pain associated with TA-TAVI. The insertion of the catheter after the implantation could be useful to avoid its displacement during the surgery.

## Background

Transapical transcatheter aortic valve implantation (TA-TAVI), in contrast to the transfemoral approach, requires special considerations for pain management because of the site of incision. Several techniques, such as epidural anesthesia and thoracic paravertebral nerve block, have been advocated [1–5], because it has been shown that the postoperative pain management by such techniques improves the postoperative morbidity and mortality [6, 7]. However, the indications for these techniques are still under debate, because the insertion of a needle into the deep structures may increase the risk of complications [8–11], especially in patients receiving antiplatelet or anticoagulant therapy, which is commonly administered in elderly patients undergoing TAVI. To avoid the complications associated with deep needle insertion, PECS II block has been suggested to be a useful option, as it is categorized as a “superficial block.” Recently, the efficacy of PECS block by bolus injection for TA-TAVI was reported [12], but the effectiveness of catheterization for continuous PECS II block has not yet been reported on postoperative pain in patients undergoing TA-TAVI. Since we considered that continuous PECS II block could be used not only intraoperatively, but also postoperatively, we report two cases undergoing TA-TAVI in which we performed continuous PECS II block for perioperative pain management.

## Case presentation

### Case 1

The first patient was a 95-year-old male with a height of 156 cm and body weight of 55 kg; he was receiving medications for hypertension. His preoperative evaluation for spinal canal stenosis revealed a cardiac murmur, which was diagnosed by transthoracic echocardiography (TTE) to be related to the presence of moderate to severe aortic stenosis (AS) (aortic valve area (AVA) 0.99 cm^2^, Vmax 3.87 m/s, peak pressure gradient (PG) 60 mmHg, mean PG 29 mmHg). Although he had no apparent symptoms, TAVI for AS was scheduled prior to the orthopedic surgery, considering his age and underlying chronic kidney disease. His AS was temporarily relieved by aortic valvuloplasty (BAV), and simultaneously, the stenosis in the left anterior descending artery (LAD) and right coronary artery (RCA) was treated by percutaneous coronary intervention (PCI) with drug-eluting stents (DES). One month after the PCI, a stent was inserted into the stenotic right iliac artery caused by arteriosclerosis obliterans (ASO). Finally, 3 months after the PCI, TA-TAVI was performed. General anesthesia was induced with propofol, rocuronium, and fentanyl, and maintained with sevoflurane. Before the incision, PECS II block was performed under ultrasound guidance (EDGE; FujiFilm SonoSite, Bothell, WA, USA). First, we put the linear probe (6–15 MHz) on the lateral third of the left clavicle to obtain the sagittal view. Thereafter, the probe was moved to the caudal direction to find the fourth rib. After identifying the pectoralis major and minor muscles and the serratus muscle at this level, the needle (Contiplex C needle; B. Braun, Melsungen, Germany) was inserted using in-plane technique until its tip reached the plane between the minor pectoralis muscles and the serratus muscle. At this plane, 24 mL of the following local anesthetic mixture (0.75% ropivacaine 20 mL, 1% mepivacaine 20 mL, and 0.9% saline 20 mL, i.e., 0.25% ropivacaine and 0.33% mepivacaine) was injected and subsequently the catheter for postoperative pain management was inserted. The intraoperative course was uneventful. The thoracotomy and the insertion of the chest drainage tube were performed at the fifth and sixth intercostal levels, respectively. Eighty-five minutes after the bolus injection, continuous infusion of 0.2% ropivacaine (infusion rate 6 mL/h, total filling amount 300 mL, Rakuraku fuser 300 FC PCA; Smiths Medical Japan Ltd., Tokyo, Japan) was started without a prior bolus injection.

**Fig. 1:**
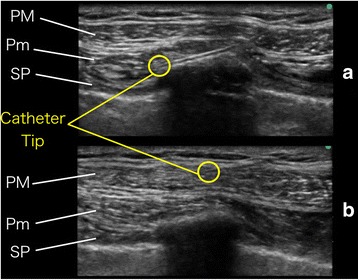
Ultrasound images related to PECS block. **a** An image just after the catheterization was obtained with catheter tip being located between the pectoralis minor and the serratus muscles on forth rib. **b** A postoperative image was obtained with displacement of the catheter tip deep in the pectoralis major muscles. PM pectoralis major muscle, Pm pectoralis minor muscle, SP serratus muscle

After the extubation, the patient was admitted to the ICU. Forty-five minutes after admission to the ICU (3 h after the bolus injection), he began to complain of severe pain. The attempt to relieve the pain by intravenous administration of buprenorphine 0.1 mg proved unsuccessful. The subsequent ultrasound examination revealed displacement of the catheter, with its tip deep in the pectoralis major muscle (Fig. 1); therefore, the continuous infusion was stopped and the catheter was removed. The pain treatment was switched to continuous infusion of fentanyl 15 mcg/h. He was transferred from the ICU to the ward on POD 1.

### Case 2

The second patient was an 87-year-old female with a height of 135 cm and body weight of 37 kg. A medical examination for her dyspnea revealed severe AS (AVA 0.46 cm^2^, Vmax 5.66 m/s, peak PG 128 mmHg, mean PG 79 mmHg by TTE). Palliative BAV had not dramatically reduced the severity of the AS, although the valve parameters were slightly improved (AVA 0.61 cm^2^, Vmax 4.01 m/s, peak PG 64 mmHg, mean PG 38 mmHg by TTE). Furthermore, the patient had underlying Alzheimer’s dementia and a tortuous descending aorta; therefore, TA-TAVI was scheduled. The anesthesia was induced and maintained using the same drugs as in the first case. Before the incision, PECS II block was performed under ultrasound guidance. The procedure of the block was the same as in the first case except for using a Tuohy needle (Arrow Epidural Needle; Teleflex, USA) for a bolus injection of 20 mL 0.375% ropivacaine and not for inserting a catheter.

**Fig. 2:**
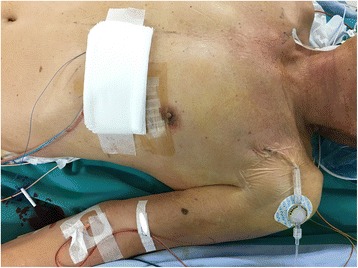
The picture shows the insertion site and the surrounding structures. Note that the catheter was placed after the operation

The thoracotomy and the insertion of the chest drainage tube were performed at the same intercostal levels as in case 1. After the implantation of the valve (3 h after the previous block), the PECS II block was repeated with a bolus injection of 20 mL 0.2% ropivacaine, and subsequently, a catheter was inserted for postoperative pain management (Contiplex C needle, B. Braun, Melsungen, Germany) (Fig. 2). Thereafter, continuous infusion was started as in case 1 before the patient was extubated and transferred to the ICU, where high-quality pain control was obtained without need for additional analgesia, although the patient had nausea, which was treated by intravenous injections of 10 mg metoclopramide. She was transferred from the ICU to the ward on POD 2.

### Discussion

From our experience in the two cases reported above, two points are noteworthy: Firstly, continuous PECS II block may have the potential to control postoperative pain in patients undergoing TA-TAVI. Secondly, the insertion of the catheter after the implantation could be a useful option to avoid its displacement due to the  distortion of the adjacent structures by the rib retractor.

In thoracic surgeries, the optimal pain management is an important aspect that determines the clinical outcomes [4, 6, 7]. Under ultrasound guidance, PECS II block can give the blockade of the lateral branch of the T2-6 spinal nerves, and possibly the anterior branch if sufficient local anesthetic agent infiltrates the external intercostal muscles. It can provide analgesia of the anterior and lower lateral thoracic wall [13, 14], which was required for the pain management of the thoracotomy and the insertion of the chest drainage tube. PECS II block is categorized as a “superficial block,” where, in contrast to the case in epidural analgesia, blockade of the sympathetic nerves is avoided. Its procedural safety owing to its technical simplicity may give the evangel, especially to patients receiving antiplatelet or anticoagulant therapy, which is commonly undertaken in elderly TAVI candidates. Although serratus plane block may also provide the analgesia for TA-TAVI from a viewpoint of the area of it, it is occasionally difficult to identify the deep side of the serratus muscle in elderly patients. We chose PECS II block because even in such cases, this block could be executed.

This method might be promising, because at least the high-quality analgesia and hemodynamic stability was observed during the operation in both the patients although in the first case, the severe postoperative pain led us to find the displacement of the catheter tip. While there is a possibility that the tip of catheter was displaced by postoperative patient’s movement or position change after the operation, we speculate that this was caused due to the distortion of the structures near the catheter by the rib retractor during thoracotomy. Therefore, in the second case, the catheterization for continuous PECS II block was performed after implantation of the valve to avoid the possible migration of the catheter tip due to distortion of the tissues. A postoperative ultrasound examination revealed that the position of the catheter tip had not changed, and high-quality pain control was maintained, not only intraoperatively, but also postoperatively. Even if a catheter should be inserted after the implantation, it might happen to migrate. Therefore, it is still important to check the position of the catheter tip by ultrasound examination in patients when continuous PECS II could not provide sufficient analgesia [15].

In case 1, a bolus PECS II block was effective for 3 h. Previous reports described the effective time of a bolus PECS II block. Blanco et al. and Kulhari et al. showed that the effective time was 8 h [14] and 294.5 min [16], respectively. We speculate that ours was shorter than theirs by two reasons. One is that the former report used a more potent dose of levobupivacaine (0.25%). It was shown that the effective time was shorter in ropivacaine than in levobupivacaine even when the clinically equipotent dose was used [17]. The other is that the latter report used ropivacaine at high concentration (0.5%). We observed the patients with caution after the block, and neither patient developed any complications.

We used catheter over needle to acquire the stability of the position [18] in the first case, but further investigations would be required to determine whether the displacement could occur depending on the type of the catheters.

## Conclusion

Continuous PECS II block may have the potential to control the perioperative pain associated with TA-TAVI. Insertion of the catheter after the implantation can be a useful option to avoid its displacement due to the distortion of the adjacent structures by the rib retractor.
